# Surgical Management of Urachal Cancer in Germany: A Nationwide Analysis From 2006 to 2022

**DOI:** 10.1245/s10434-026-19877-7

**Published:** 2026-06-01

**Authors:** Luka Flegar, Nicole Eisenmenger, Philipp Reimold, Rainer Koch, Johannes Huber, Christer Groeben

**Affiliations:** 1 Department of Urology, University of Heidelberg, Heidelberg, Germany; 2 Reimbursement Institute, Hürth, Germany

**Keywords:** Urachal cancer, Minimal-invasive surgery, Health services research, Robotics, Population-based analysis

## Abstract

**Objective:**

Urachal cancer is a rare but highly aggressive malignancy. This study examined current trends in the surgical management of urachal cancer in Germany.

**Material & Methods:**

The nationwide German hospital billing database (Destatis) and German quality reports were analyzed from 2006 to 2022. Identification of trends over time was performed using linear regression models.

**Results:**

Between 2006 and 2022, a total of 2593 cases were treated for urachal cancer in Germany. In 530 cases, a partial (*n* = 413) or radical (*n* = 117) cystectomy was performed. The share of partial cystectomy for treatment of urachal cancer increased from 52% in 2006 to 74% in 2022 (p<0.001). Radical cystectomy decreased from 48% in 2006 to 26% in 2022. Robotic-assisted partial cystectomy increased slightly from 9.7% in 2019 to 10.5% in 2022. The median age of patients undergoing partial cystectomy for urachal cancer increased from 57 ± 15.35 years in 2006 to 62 ± 12.59 years in 2022 (p<0.001). Median length of stay after open partial cystectomy decreased from 14 days in 2006 to 9 days in 2022 (p<0.001). Median length of stay for robotic-assisted partial cystectomy was 7 days in 2022. The mortality rate following partial cystectomy was 0.753%. The most common in-hospital complications associated with urachal cancer were hematuria in 1.19%, severe hemorrhage and hematoma in 0.96%, and paralytic ileus in 0.61%.

**Conclusion:**

This study demonstrates an increasing trend toward the use of partial cystectomy in the treatment of urachal cancer, along with a trend toward shorter hospital stays in recent years.

**Supplementary Information:**

The online version contains supplementary material available at 10.1245/s10434-026-19877-7.

Urachal cancer is a rare and aggressive malignancy that arises from remnants of the embryonic urachus, presenting distinct challenges in both diagnosis and treatment.^1^ Accounting for less than 1% of all bladder cancers, urachal cancer predominantly affects adults in their fifth or sixth decade of life.^2^ Adenocarcinoma is the most prevalent histological type of urachal cancer. Hematuria commonly constitutes the primary symptom and mostly a transurethral resection of a bladder tumor is performed for diagnostics.^3^ However, it can also be associated with nonspecific symptoms and so is often diagnosed only at advanced stages.^4^ Surgical resection, typically through partial or radical cystectomy with en bloc removal of the urachus and umbilicus, remains the gold standard.^5,6^ Nonetheless, variations in surgical techniques, oncological outcomes, and patient-specific factors emphasize the importance of thoroughly understanding evolving trends in clinical management.^6^ International guidelines generally recommend partial cystectomy to prioritize organ preservation and enhance quality of life, with a 5-year overall survival rate of approximately 50%.^5,7^

Over the past 2 decades, new minimally invasive surgical techniques, advancements in imaging, and the centralization of cancer care have likely influenced the management of urachal cancer.^5,8^ Despite these developments, population-based analyses remain limited.

Therefore, this study aims to analyze trends in the surgical management of urachal cancer in Germany between 2006 and 2022.

## Materials and Methods

For the present analysis, datasets from the quality reports from German hospitals and from the German Billing Database (Destatis) were analyzed. Table S1 in the supplementary material gives a detailed overview of the queried databases.

The Destatis database allows the combined analysis of International Classification of Diseases and Related Health Problems, Tenth Edition (ICD-10) codes and surgical procedure codes (OPS codes [Operationen und Prozedurenschluessel; –the German version of the ICD). Thus, these datasets were used for analysis of all surgical procedures. The quality reports provide geographical information on surgical treatments or medical diagnoses and were therefore used to identify national providers. The data extraction and cohort identification methods are described in previous studies from our research group.^9^ Inclusion criteria were age at least 18 years.

For analysis of urachal cancer, the ICD code “c.67.7” was studied. Further we examined the OPS codes 5.575 and 5.576, representing “partial cystectomy”, or 5.687 representing “radical cystectomy” (in males or females, respectively). In addition, the OPS code 5.987 for “robotic-assisted procedures” was analyzed. By combining the ICD code “c.67.7” with the OPS codes 5.575 and 5.576 and the OPS code 5.987, we were able to study all robotic-assisted partial cystectomies for urachal cancer within the analyzed period. To detect the most common in-hospital complications associated with urachal cancer, various ICD codes were analyzed (Table S2 in the supplementary material). Further, procedures (OPS codes) associated with complications were assessed. Additionally, to study complications and their treatments in greater detail from 2006 to 2022, we defined three categories of complications (Table S2 in the supplementary material). The first category focused on ileus and its associated therapeutic interventions. The second examined hematuria and related treatments, such as continuous irrigation. The third addressed surgical revisions, including those for hematoma or wound dehiscence.

### Statistical Analysis

Data are presented using absolute and relative frequencies. For identification of trends over time, linear regression models were utilized. For calculations, the chi-squared test was performed. Statistical significance was defined as p<0.05. SPSS version 28 (IBM Corp., Armonk, NY, USA) was used for calculations and the statistical analysis.

### Results

Between 2006 and 2022, a total of 2593 procedures for urachal cancer in Germany were identified. In 530 cases (20.4%) a partial (*n* = 413) or radical (*n* = 117) cystectomy was performed.

In 2006, a total of 52 hospitals treated patients with urachal cancer, and this increased to 98 hospitals in 2022. Four of 52 hospitals (7.7%) treated more than three patients in 2006, and six of 98 hospitals (6.1%) treated more than three patients in 2022.

**Fig. 1:**
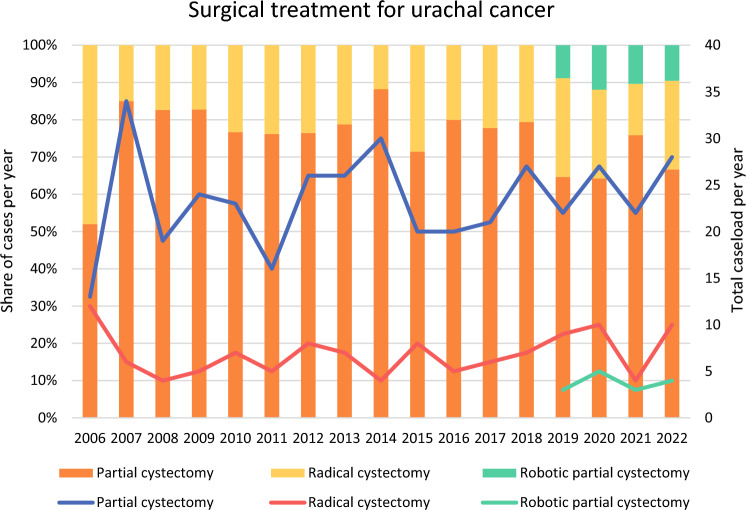
Share of performed treatment of urachal cancer in Germany from 2006 to 2022 (source: Destatis database)

The share of partial cystectomy for treatment of urachal cancer increased from 52% in 2006 to 74% in 2022 (p<0.001). Radical cystectomy decreased from 48% in 2006 to 26% in 2022. Robotic-assisted partial cystectomy increased slightly from 9.7% in 2019 to 10.5% in 2022 (Fig. 1). Partial cystectomy for urachal cancer rose from 13 cases in 2006 to 28 in 2022 (p<0.001), whereas radical cystectomy remained stable (12 vs. 10 cases). Robotic-assisted partial cystectomy increased modestly, reaching four cases in 2022 (Fig. 1).

In 2022, 38% of performed cases with urachal cancer were in women, and 62% were in men. The median age of patients undergoing partial cystectomy for urachal cancer increased from 57 ± 15.35 years in 2006 to 62 ± 12.59 years in 2022 (p<0.001); for patients undergoing radical cystectomy, the median age increased from 64.5 ± 15.45 years to 67 ± 9 years in 2022 (p<0.001).

**Fig. 2:**
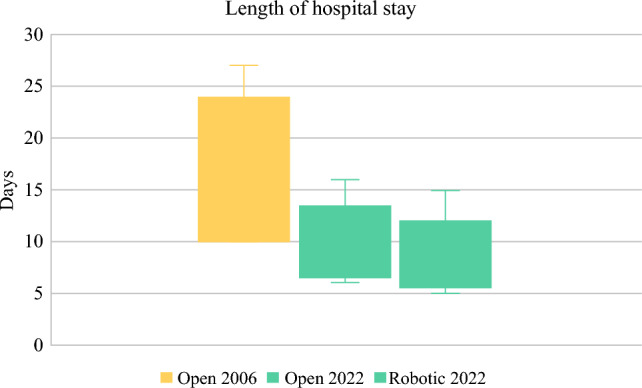
Length of hospital stay for partial cystectomy for urachal cancer in Germany in 2006 and 2022 (source: Destatis database)

Figure 2 displays the median length of stay (LOS) after open partial cystectomy for urachal cancer, which decreased from 14 days in 2006 to 9 days in 2022 (p<0.001). The median LOS for robotic-assisted partial cystectomy was 7 days in 2022.

The overall mortality rate for surgical treatment was 0.753% (3/398). These were all attributable to open partial cystectomy.

The most common in-hospital complications associated with urachal cancer were persistent hematuria in 1.2%, severe hemorrhage and hematoma in 0.96%, and paralytic ileus in 0.61%. Table 1 displays the 10 most common in-hospital complications and procedures associated with urachal cancer. Continuous irrigation was required in 3.3% of cases, whereas a vacuum-assisted closure (VAC) was placed in 1.4% of cases, and a secondary closure of the abdominal wall (in cases of postoperative wound dehiscence) was needed in 1.3% of cases. Table 2 represents the case numbers for treatment of different complications after urachal cancer surgery from 2006 to 2022.

**Table 1:** The ten most frequent in-hospital complications and procedures associated with urachal cancer from 2006 to 2022

	ICD code	Complication	Patients	OPS code	Procedure	Patients
1	R31	Hematuria	31 (1.2)	8-132.3 8-132.2 8-132.1 8-132.x 8-132.0	Continuous irrigation	85 (3.3)
2	T810	Hemorrhage and hematoma complicating a procedure, not elsewhere classified	25 (0.96)	5-916a3 8-190.23 8-190.13	Placement of VAC	36 (1.4)
3	K560	Ileus paralytic	16 (0.61)	5-545.0 5-900.1b	Secondary closure of the abdominal wall (in cases of postoperative wound dehiscence)	33 (1.3)
4	N300	Acute cystitis	14 (0.54)	1-632.0	Gastroscopy	29 (1.1)
5	T835	Infection and inflammatory reaction due to prosthetic device, implant, and graft in urinary system	14 (0.54)	5-455.71 5-455.01 5-455.41	Partial colectomy	22 (0.84)
6	N183	Chronic kidney disease, stage 3	14 (0.54)	5-983	Surgical revision	20 (0.77)
7	N302	Chronic cystitis	11 (0.42)	1-650.1	Coloscopy	18 (0.69)
8	G470	Disorders of initiating and maintaining sleep	11 (0.42)	5-541.2	Relaparotomy	12 (0.46)
9	R140	Abdominal distension (gaseous)	9 (0.34)	8-176.2	Open abdominal lavage	11 (0.42)
10	R33	Retention of urine	8 (0.30)	5-462.1	Ileostomy	6 (0.23)

Data are presented as n (%) unless otherwise indicated

ICD, International Classification of Diseases and Related Health Problems; OPS, Operationen und Prozedurenschluessel (the German version of the ICD)

**Table 2:** The ten most frequent in-hospital complications and procedures associated with surgical cases (3 blocks) due to complications from 2006 to 2022

	2006	2007	2008	2009	2010	2011	2012	2013	2014	2015	2016	2017	2018	2019	2020	2021	2022
Bowel complications	4	6	0	0	6	7	3	4	3	9	5	4	5	7	0	4	6
Postoperative bleeding/hematuria	13	13	4	11	10	7	12	12	11	11	10	9	10	15	13	8	11
Surgical revision	0	7	0	0	5	3	4	3	5	3	0	0	8	3	3	4	6

## Discussion

The present population-based study examined current trends in the surgical management of urachal cancer in Germany. Several key findings were observed. Partial cystectomy for surgical management of urachal cancer increased in recent years and now represents the primary treatment choice. Although the median age of patients undergoing surgical treatment for urachal cancer increased, LOS significantly decreased during the examined study period.

### Diagnosis and Treating Hospitals

We identified 2593 cases with urachal cancer between 2006 and 2022. Hager et al.^10^ showed similar results with only 154 cases reported cases between 2011 and 2015. An Irish study from 2016 highlighted the need for centralization of rare diseases such as urachal carcinomas to improve overall patient survival.^11^ Further, the authors recommended an international collaboration to identify optimal treatment strategies. In the case of rare cancers such as urachal cancer, high-volume centralized centers are better positioned to gain experience, build expertise, and optimize treatment approaches, leading to improved long-term outcomes for patients. The number of hospitals treating urachal cancer nearly doubled from 52 in 2006 to 98 in 2022, without clear evidence of a trend toward centralization. The proportion of hospitals managing more than three cases annually remained low (7.7% in 2006 vs. 6.1% in 2022), underscoring the rarity of the disease and the potential benefits of further centralizing care in high-volume centers.

### Surgical Trends

The share of partial cystectomy increased significantly from 52% in 2006 to 74% in 2022. A recent population-based study from the USA compared radical and partial cystectomy. The authors showed that the radical approach was not associated with a lower cancer-specific mortality. They concluded that partial cystectomy should be performed as primary treatment unless contraindicated because of either extension of the primary tumor, an insufficient bladder capacity, or other severe dysfunctions of the lower urinary tract.^12^ Chen et al.^13^ discussed the importance of negative surgical margins after partial cystectomy providing outcomes comparable to those with radical cystectomy. The proportion of robotic-assisted partial cystectomy increased modestly from 9.7% to 10.5%, remaining a small but emerging share of cases, with three procedures performed in 2019 and four in 2022. James et al.^14^ showed that robotic-assisted partial cystectomy was a feasible and safe procedure for treatment of urachal cancer. This shift aligns with broader trends in urologic oncology, where robotic surgery is associated with reduced morbidity and shorter hospital stays.^9,15^ Current evidence suggests that oncologic outcomes, particularly positive surgical margin rates, are broadly comparable across open, laparoscopic, and robotic approaches, although data remain limited and heterogeneous. In the largest comparative urachal series, positive surgical margin rates were similar between open and robotic partial cystectomy (~13%), whereas robotic platforms may show improved margin control over time due to enhanced visualization and surgeon learning curves.^16^ Meanwhile, radical cystectomy numbers decreased to 26% in 2022. Our working group previously analyzed radical cystectomy for muscle-invasive bladder cancer in Germany. We showed a constant share of patients receiving radical cystectomy for muscle-invasive bladder cancer between 2006 and 2019.^17^

### Patient Demographics and Length of Stay

The median age of patients undergoing surgery for urachal cancer increased significantly. Patients undergoing partial cystectomy were younger (62 ± 12.59 years in 2022) than those undergoing radical cystectomy (67 ± 9 years in 2022), reflecting possible differences in patient selection criteria and disease severity. Urachal cancer typically presents at a median age of 52–59 years, making it a disease that generally affects younger patients compared with urothelial bladder cancer.^3,5^ Further, two of three patients diagnosed with urachal cancer were men. These results are in line with those from international studies.^3,18^

Notably, the LOS after partial cystectomy decreased from 14 days in 2006 to 9 days in 2022, highlighting advancements in perioperative care and recovery protocols. Robotic-assisted partial cystectomy had an even shorter LOS of 7 days, further supporting its potential benefits. Previous studies showed shorter LOS for robotic-assisted procedures.^9,19^ Overall, the in-hospital complication rate associated with urachal cancer was rather low. The mortality rate following open partial cystectomy (0.753%) was low; however, this finding should be interpreted with caution when considering the safety and efficacy of this surgical approach. No deaths were associated with robotic-assisted partial cystectomy. Continuous irrigation and VAC placement were the most common procedures associated with complications after urachal cancer treatment. Overall complication rates were low, reflecting the bladder-sparing nature of partial cystectomy. In contrast, radical cystectomy, typically performed for muscle-invasive bladder cancer, carries higher risks of hemorrhage, infection, ileus, urinary diversion-related complications, and longer recovery.^17,20^

## Limitations

This study is the first to provide German population-based data on urachal cancer treatment over a 16-year period, but several limitations must be acknowledged. Although billing data can be regarded as highly accurate, the retrospective nature of the data and reliance on hospital records may limit our findings. Repeat admissions or duplicate cases may introduce bias and attenuate the observed effects. Further clinical information on disease staging, histopathological subtypes, and adjuvant treatments was not available in the datasets. Moreover, surgical revision or readmission due to late complications could only be assessed during the same hospital stay. Because single cases or hospitals may not be identified from the database, it was not possible to verify whether each dataset had been entered correctly and according to standards. Our findings are primarily descriptive and do not allow for a definitive assessment of whether the observed shift toward partial cystectomy is oncologically appropriate. Further, the mortality rates are likely underestimated since our analysis is restricted to in-hospital events. Finally, outpatient treatments for urachal cancer were not included in the databases.

## Conclusion

This study demonstrates a growing preference for partial cystectomy in the treatment of urachal cancer, along with a trend toward shorter hospital stays in recent years. No clear evidence of centralization in the management of this rare disease was observed. Additionally, our results suggest potential gaps in nationwide coverage within Germany.

## Supplementary Information

Below is the link to the electronic supplementary material.

Supplementary file1 (DOCX 83 KB)

## Data Availability

German research data center of the federal statistical office, DRG statistics 2006–2022, German “National Centre for Cancer Registry Data” (Robert Koch Institute, Berlin), own calculations. German hospital quality reports are publicly accessible. The datasets generated during and/or analyzed during the current study are available from the corresponding author on reasonable request.
