# Supplementary material for: Surgical Management of Urachal Cancer in Germany: A Nationwide Analysis From 2006 to 2022
Source: Ann Surg Oncol. 2026 Jun 1;33(9):8630–5. doi: 10.1245/s10434-026-19877-7 (PMC13452856; doi:10.1245/s10434-026-19877-7)
Supplement: Supplementary file 1 [file 10434_2026_19877_MOESM1_ESM.docx]

**Supplement**

**Table 1.** Analyzed databases.

| **Data source** | Nationwide hospital billing database of the German Federal Statistical Office  (Destatis database) | German hospitals’ quality reports  (reimbursement.INFO tool) |
| --- | --- | --- |
| **Data details** | - Age and gender - Diagnosis code - Type of surgery and approach - Hospital characteristics (teaching status, size, annual surgery caseload, approaches for surgery) - Patients age ≥18 | - Age and gender - Type of surgery - Hospital characteristics (teaching status, annual surgery caseload) - Geographical localization of respective hospitals - Patients age ≥20 |
| **Data query option** | - Combination of OPS- and ICD- code possible | - Only OPS- or ICD-code |
| **Number of patients** | 2593 | 1818 |
| **Proportion of the country** | 100% | 100% |
| **Included years** | 2006 - 2022 | 2006, 2008, 2010, and 2012-2022 |

**Table 2.** Surgical codes

**Bowel complications:**

**ICD_C677 AND (OPS_5575 OR OPS_5576 OR OPS_5687) AND ((ICDND_K560 OR ICDND_R140) OR (OPS_1653 OR OPS_545541 OR OPS_54621 OR OPS_545460 OR OPS_545501 OR OPS_545571 OR OPS_1632 OR OPS_1440a OR OPS_16501))**

**Post operative bleeding / hematuria:**

**ICD_C677 AND (OPS_5575 OR OPS_5576 OR OPS_5687) AND ((ICDND_R31 OR ICDND_T810) OR (OPS_8132 OR OPS_8800))**

**Surgical revision:**

**ICD_C677 AND (OPS_5575 OR OPS_5576 OR OPS_5687) AND (OPS_5916a3 OR OPS_5983 OR OPS_55450 OR OPS_59001b OR OPS_55410 OR OPS_55412 OR OPS_81762 OR OPS_55411 OR OPS_819023 OR OPS_819013 or OPS_81440)**
